# Supplementary material for: A PIANO (Proper, Insufficient, Aberrant, and NO Reprogramming) Response to the Yamanaka Factors in the Initial Stages of Human iPSC Reprogramming
Source: Int J Mol Sci. 2020 May 2;21(9):3229. doi: 10.3390/ijms21093229 (PMC7246695; doi:10.3390/ijms21093229)
Supplement: Supplementary file 1 [file ijms-21-03229-s001.zip › ijms-781518-supplementary-for conversion/ijms-781518-supplementary-for conversion.pdf]

Supplementary:

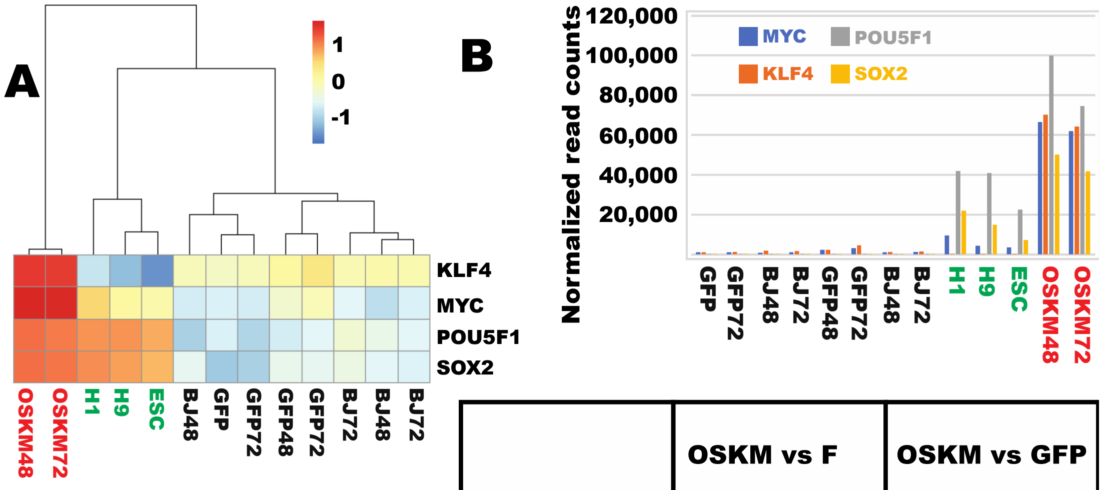

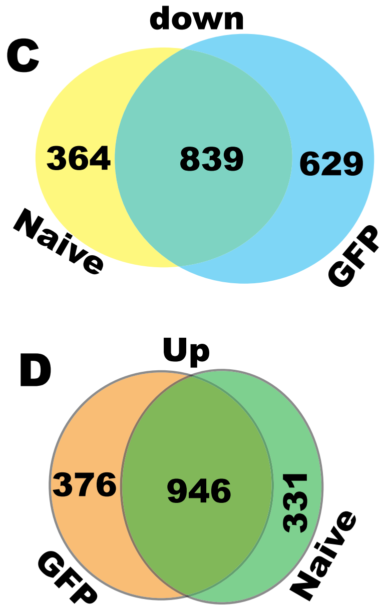

E

		OSKM vs F	OSKM vs GFP
Hu	Up	1277	1322
	Down	1203	1469
	Total	2480	2791
	Criteria	2x, q < 0.01	2x, q < 0.01
Mah	comon total	679	Not tested
	Criteria	1.5x, q < 0.05	NA

Supplementary Figure S1. Summary of the OSKM ectopic expression in human fibroblasts in this study. A, Heat map showing successful expression of OSKM in comparison with those in ESCs (n = 3), naïve human fibroblasts BJ (n = 4), and fibroblasts expressing lentiviral GFP (n = 4). B, A bar graph with the normalized read counts for samples in A. C, Venn diagram showing that 839 genes were commonly down-regulated genes at both time points (48 and 72 hours) as compared with the naïve and GFP-transduced human fibroblasts. D, Venn diagram showing 946 genes were commonly up-regulated genes at both time points (48 and 72 hours) as compared with the naïve and GFP-transduced human fibroblasts. E, The RNA-seq technology used here identified much more differentially expressed genes by OSKM than microarray technology used by Mah et al. Highlighted in red are total numbers of genes differentially expressed by OSKM at both time points (48 and 72 hours). The more stringent sorting criteria in this study are highlighted in cyan. Note that Mah et al. did not use the GFP control. OSKM, human fibroblasts transduced with OSKM lentiviruses for the period of time as indicated (48 and 72 hours). Numbers after the naïve fibroblasts, i.e., BJ48 and

BJ72, are the RNA harvest time for a mock transduction (the same procedures but without viruses). OSKM, OCT4, SOX2, KLF4, and c-MYC. POU5F1 = OCT4.

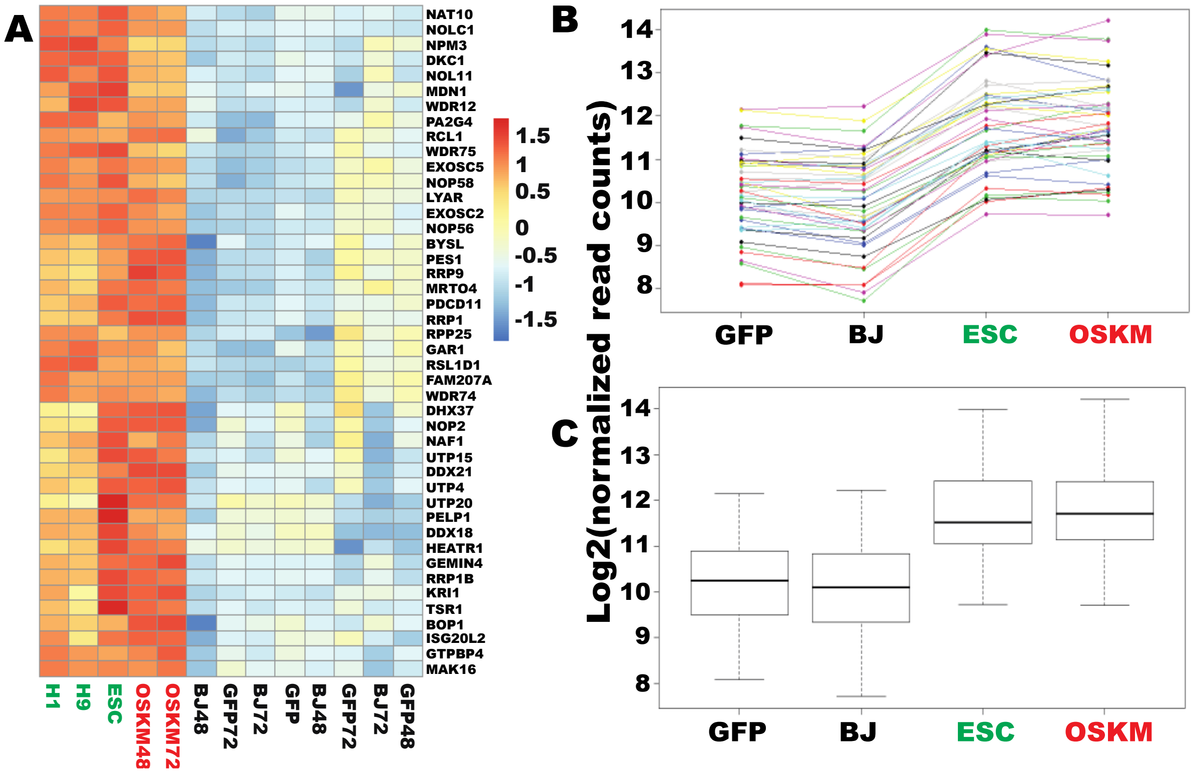

Supplementary Figure S2. 44 genes with roles in rRNA processing were successfully reprogrammed within 48 hours. A, Heat map of the 44 genes prepared with the log2-transformed normalized read counts. B, Ladder plot of the same set of data in A, but based on the average read counts. C, box plot of data in B. Figure sample labels are the same as in Figure 1.

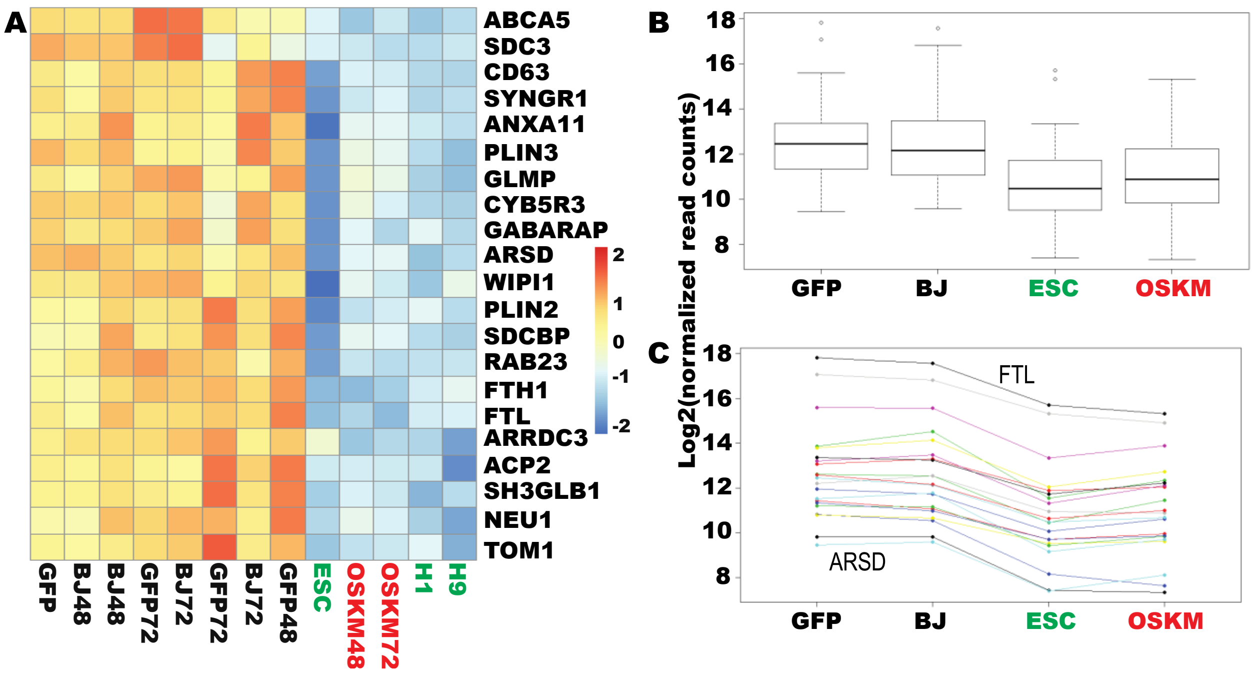

Supplementary Figure S3. 21 fibroblast-enriched genes of vacuole components were properly down-reprogrammed to the pluripotent state. A, Heat map of the 21 fibroblast-enriched vacuole genes showing expression levels before and after OSKM reprogramming. B, box plot for data in A, but with the averaged read counts. C, ladder plot for data in A, but with the averaged read counts.

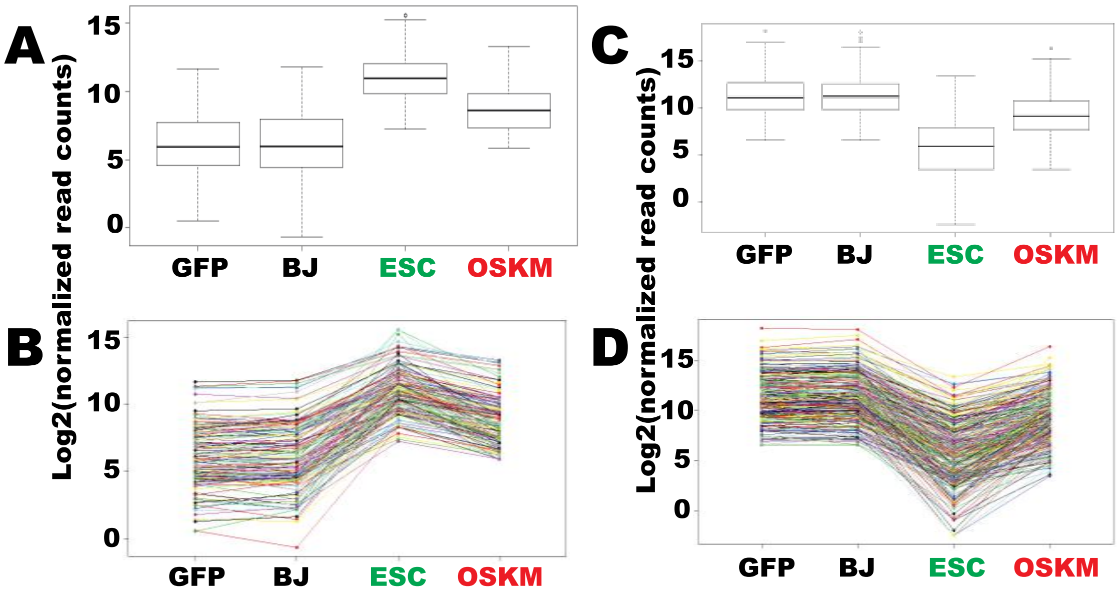

Supplementary Figure S4. Two sets of genes are significantly but insufficiently reprogrammed by OSKM at the early stages. A, A box plot showing that 152 ESC-enriched genes were significantly up-regulated but insufficiently reprogrammed by OSKM. B, A ladder plot showing that 152 ESC-enriched genes were significantly up-regulated but insufficiently reprogrammed by OSKM. C, A box plot showing that 286 fibroblast-enriched genes were significantly down-regulated but insufficiently reprogrammed by OSKM. D, A ladder plot showing that 286 fibroblast-enriched genes were significantly down-regulated but insufficiently reprogrammed by OSKM. GFP, human fibroblasts transduced with GFP lentiviral vectors (n = 4); BJ, human fibroblasts (n = 4); ESC, human embryonic stem cells (n = 3); OSKM, human fibroblasts transduced with OSKM lentiviral vectors (n = 2 at two time points, i.e., 48 and 72 hours).

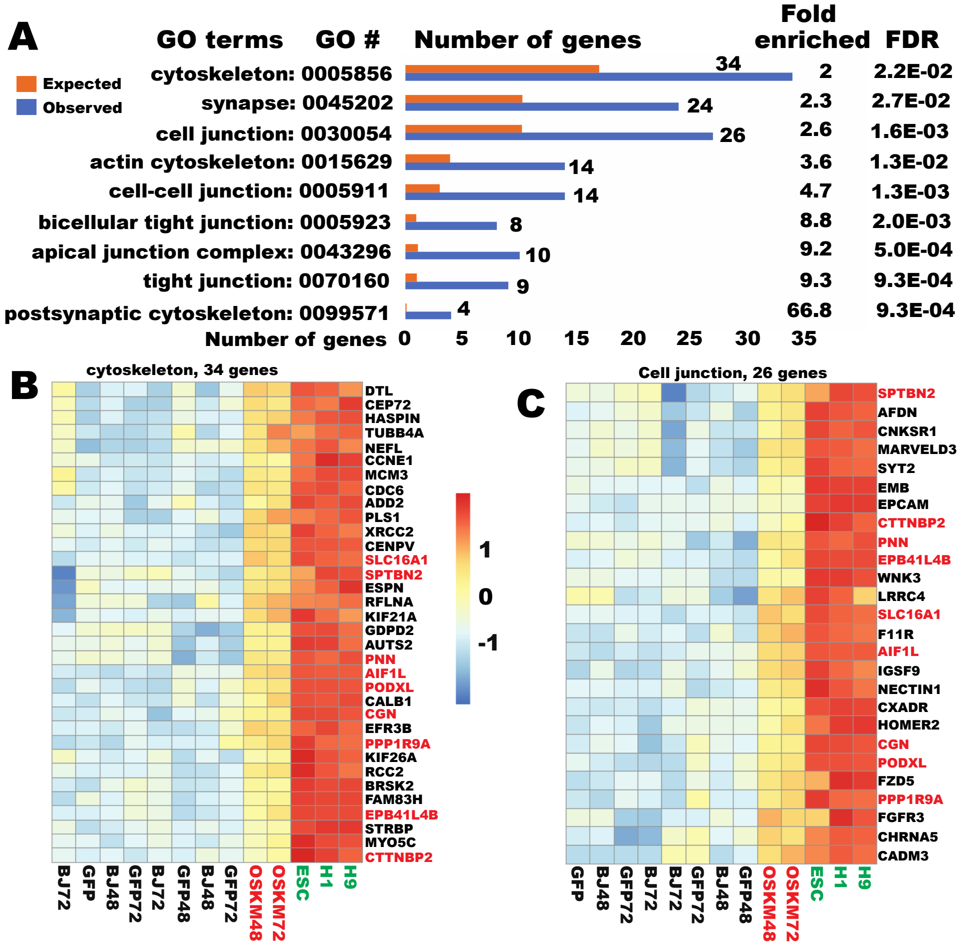

Supplementary Figure S5. A subset of genes involved in cytoskeleton and cell junction were significantly up-regulated but insufficiently up-reprogrammed. A, Summary of GO analysis of the 152 insufficiently up-reprogrammed genes with the GO annotation data set of "cellular component complete". B, A heat map showing the insufficient up-reprogramming of the 34 cytoskeleton genes as labelled. C, A heat map showing insufficient up-reprogramming of the 26 cell junction genes as indicated. The common genes (i.e., row names) between these two groups were highlighted in red. Human embryonic stem cells (i.e., column names) are highlighted in green; OSKM induction is highlighted in red; and human fibroblast samples (both naïve and GFP transduced) are in black. FDR , false discovery rate. GO, gene ontology.

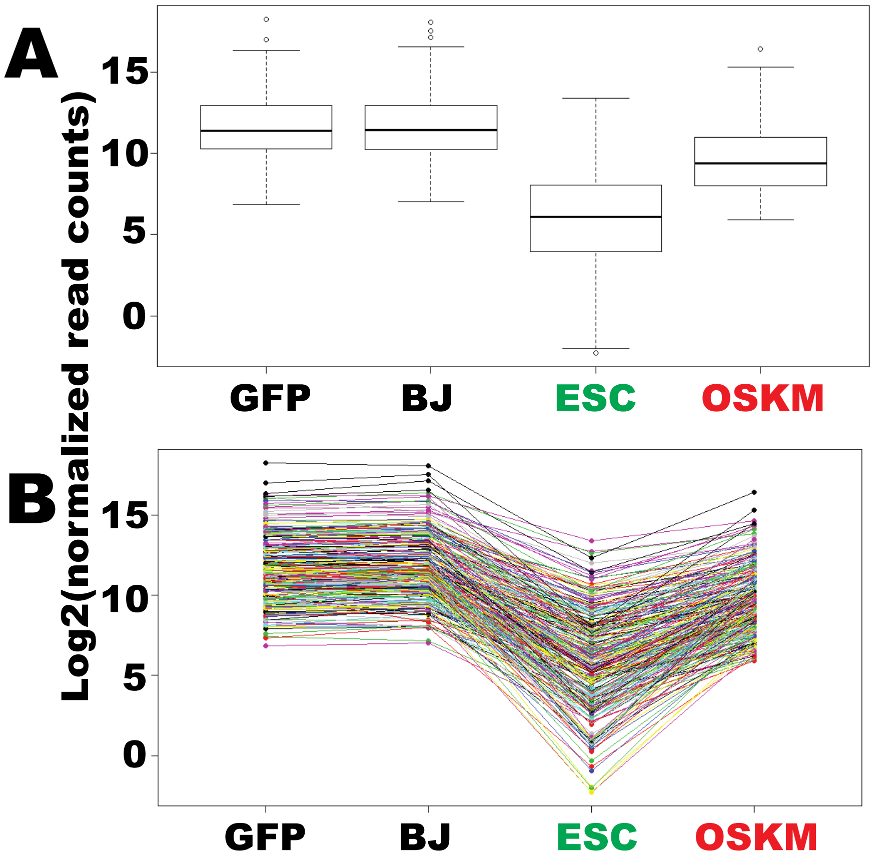

Supplementary Figure S6. 286 fibroblast-enriched genes were significantly down-regulated but insufficiently down-reprogrammed by OSKM at the early stage. A, A box plot showing significant down-regulation yet insufficient down-reprogramming of the 286 fibroblast-enriched genes by OSKM at the early stages of reprogramming. B, A ladder plot for data in A.

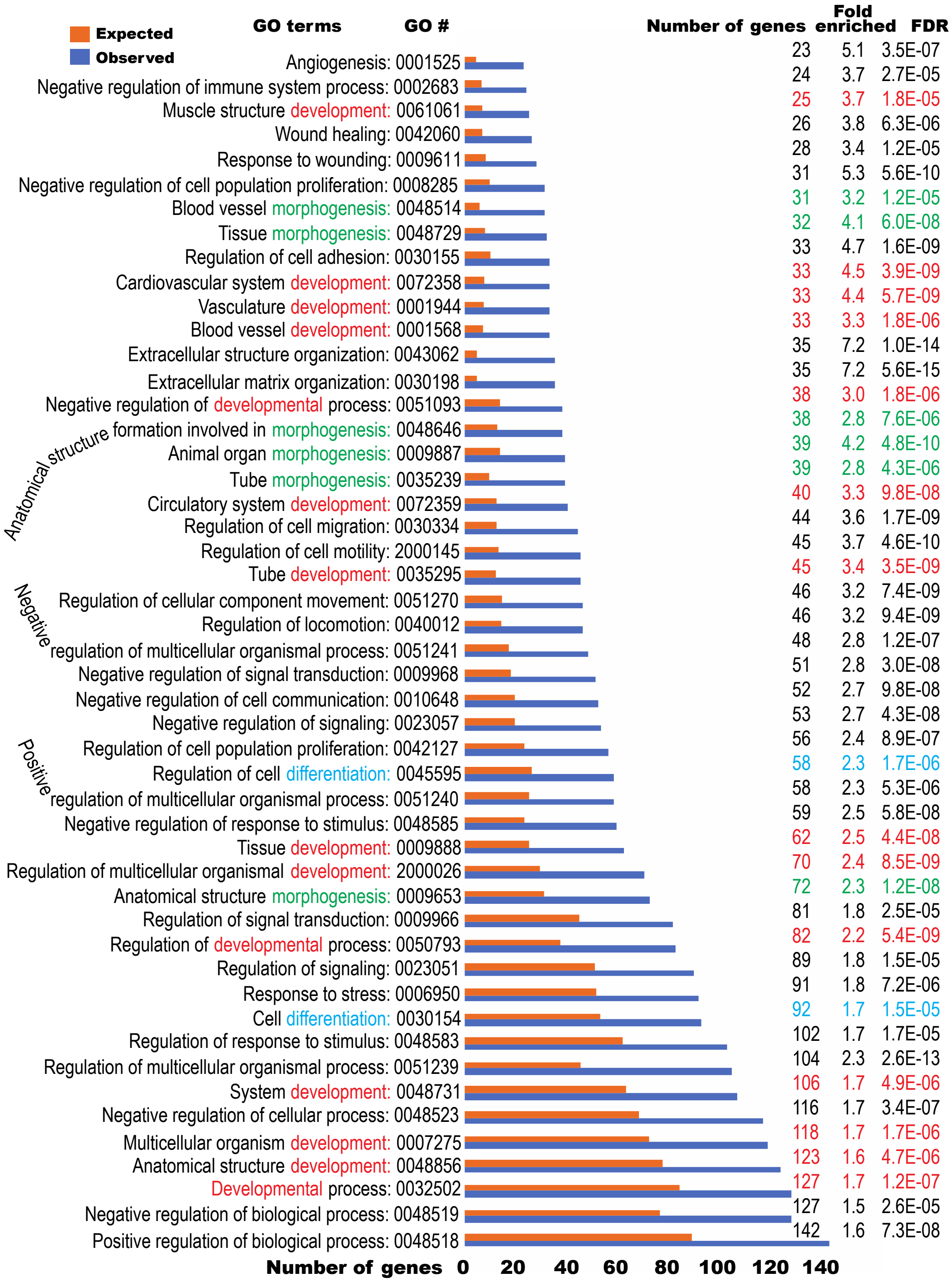

Supplementary Figure S7. Summary for the GO analysis of the 286 insufficiently down-reprogrammed fibroblast genes with the GO annotation data set of “biological process complete”. GO terms with the key word of “development” and their associated statistic data are highlighted in red; GO terms with the key word of “morphogenesis” and their associated statistic data are highlighted in green; and those with “differentiation” in cyan. FDR, false discovery rate; GO, gene ontology. Test type, Fisher’s exact.

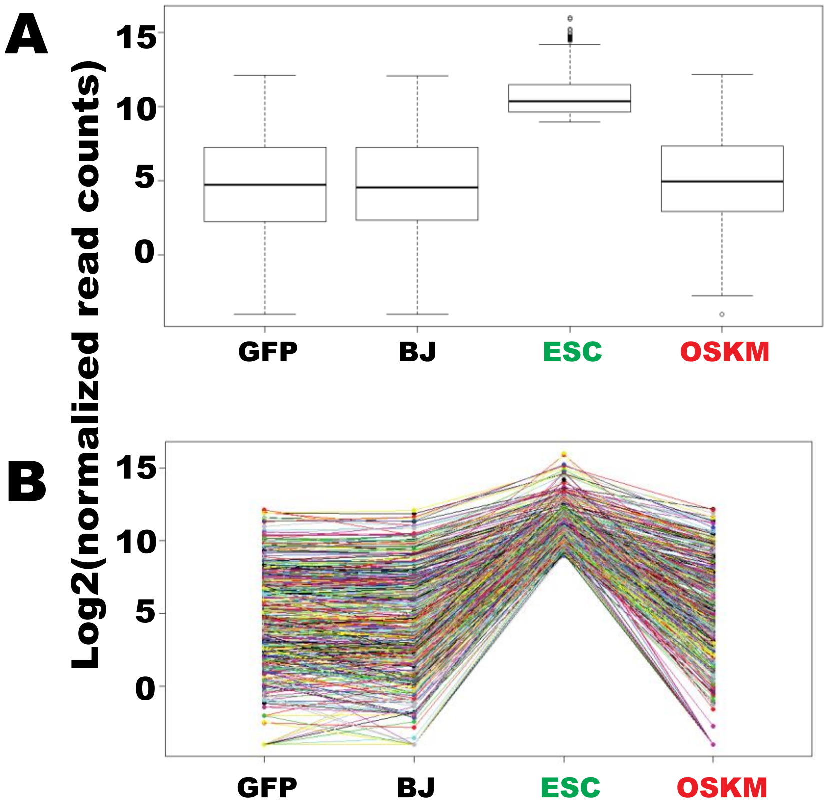

Supplementary Figure S8. 504 ESC-enriched genes are resistant to OSKM reprogramming at the early stage. A, A box plot showing that 504 ESC-enriched genes were not responding transcriptionally to OSKM regulation at the early stage. B, A ladder plot for data in A to show the irresponsiveness in a more individual way. ESC, embryonic stem cells (n = 3); BJ, human fibroblasts (n = 4); GFP, human fibroblasts transduced with GFP lentiviruses (n = 4); OSKM, human fibroblasts transduced with OSKM lentiviruses (n = 2).

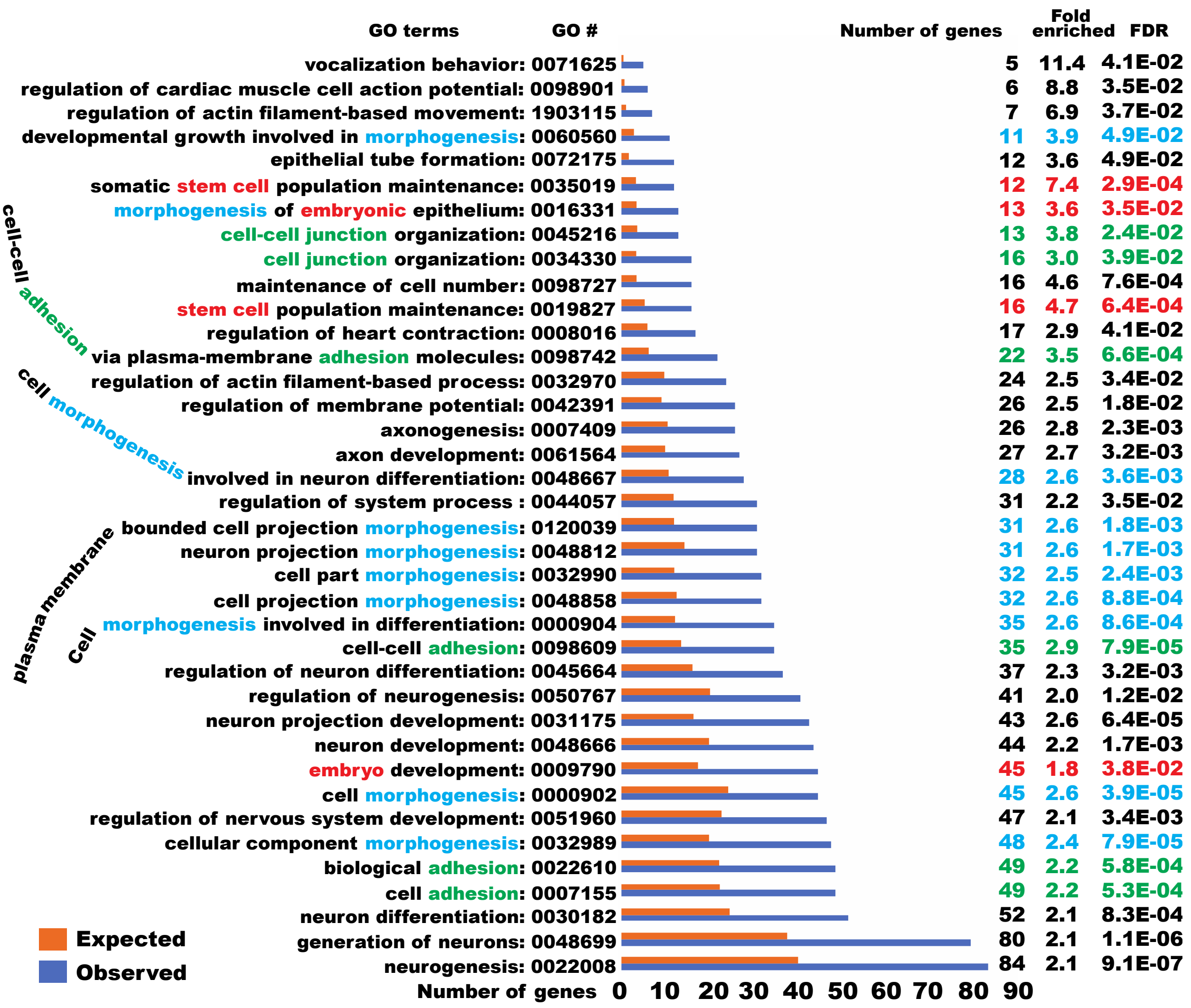

Supplementary Figure S9. Summary for GO analysis of the 504 ESC-enriched genes resistant to OSKM reprogramming. GO terms and their associated statistics with the key words of “stem cells” and “embryo” are highlighted in red; those with the key word of “morphogenesis” in cyan; those with “adhesion” or “cell junction” in green.

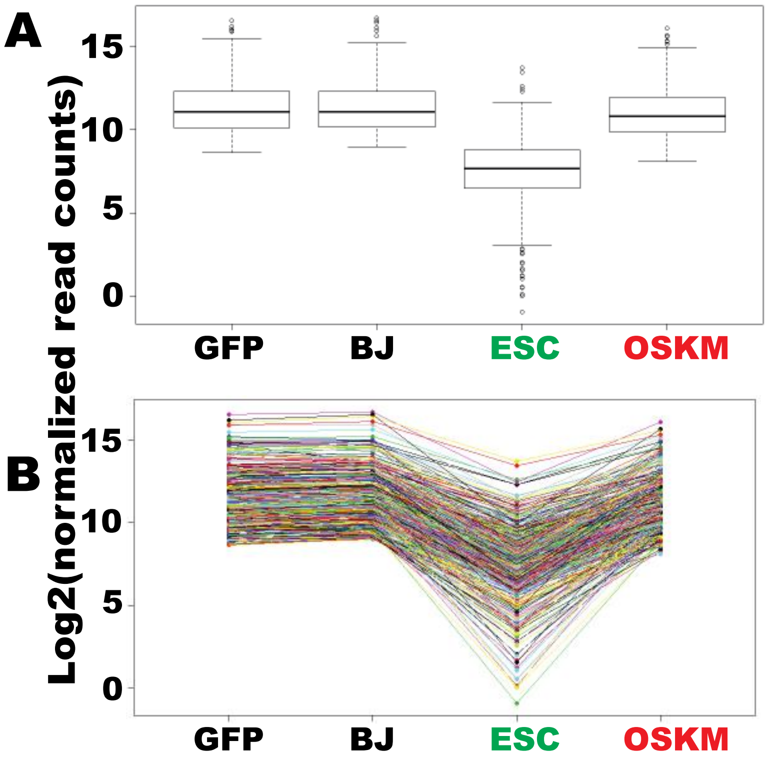

Supplementary Figure S10. 449 fibroblast-enriched genes are resistant to OSKM reprogramming at the early stage. A, A box plot showing that 449 fibroblast-enriched genes did not respond transcriptionally to OSKM reprogramming. B, A ladder plot for the data in A to show individually the irresponsiveness of the 449 genes to reprogramming. ESC, embryonic stem cells (n = 3); BJ, human fibroblasts (n = 4); GFP, human fibroblasts transduced with GFP lentiviruses (n = 4); OSKM, human fibroblasts transduced with OSKM lentiviruses (n = 2).

GO terms	GO #	Number of genes	Fold enriched	FDR
unsaturated fatty acid biosynthetic process:	0006636	3	26.9	3.4E-02
autophagosome maturation:	0097352	4	13.8	3.0E-02
substrate adhesion-dependent cell spreading:	0034446	6	12.2	3.9E-03
regulation of cell shape:	0008360	7	7.0	1.2E-02
extracellular matrix organization:	0030198	7	5.7	2.9E-02
extracellular structure organization:	0043062	7	5.0	4.4E-02
regulation of cell migration:	0030334	8	4.4	4.3E-02
positive regulation of developmental process:	0051094	8	4.3	4.5E-02
regulation of cell morphogenesis:	0022604	9	4.3	3.1E-02
circulatory system development:	0072359	9	4.3	3.2E-02
cell-substrate adhesion:	0031589	10	9.8	1.7E-04
regulation of anatomical structure morphogenesis:	0022603	12	4.1	9.1E-03
regulation of cell population proliferation:	0042127	12	4.0	1.0E-02
cell population proliferation:	0008283	12	3.5	2.3E-02
cell morphogenesis involved in differentiation:	0000904	13	3.2	3.0E-02
tissue development:	0009888	14	3.4	1.1E-02
regulation of developmental process:	0050793	15	2.7	3.9E-02
cell morphogenesis:	0000902	19	3.3	3.2E-03
cell development:	0048468	19	2.4	4.3E-02
cell migration:	0016477	20	3.5	1.0E-03
cellular component morphogenesis:	0032989	20	3.1	3.1E-03
cell motility:	0048870	20	3.1	3.0E-03
localization of cell:	0051674	20	3.1	3.3E-03
animal organ development:	0048513	20	2.8	8.0E-03
locomotion:	0040011	22	2.7	5.0E-03
biological adhesion:	0022610	24	3.6	1.4E-04
cell adhesion:	0007155	24	3.6	2.1E-04
regulation of molecular function:	0065009	30	2.0	3.6E-02
regulation of cell communication:	0010646	30	2.0	3.8E-02
regulation of signaling:	0023051	30	2.0	3.8E-02
anatomical structure morphogenesis:	0009653	32	3.1	1.3E-04
system development:	0048731	35	2.2	3.6E-03
cell differentiation:	0030154	36	2.2	3.6E-03
multicellular organism development:	0007275	37	2.1	5.8E-03
cellular developmental process:	0048869	40	2.3	1.2E-03
anatomical structure development:	0048856	49	2.2	2.4E-04
positive regulation of cellular process:	0048522	49	1.8	1.3E-02
positive regulation of biological process:	0048518	53	1.7	2.3E-02
developmental process:	0032502	54	2.1	1.6E-04
regulation of cellular metabolic process:	0031323	61	1.6	3.9E-02
regulation of cellular process:	0050794	116	1.4	2.6E-02
regulation of biological process:	0050789	119	1.3	4.3E-02

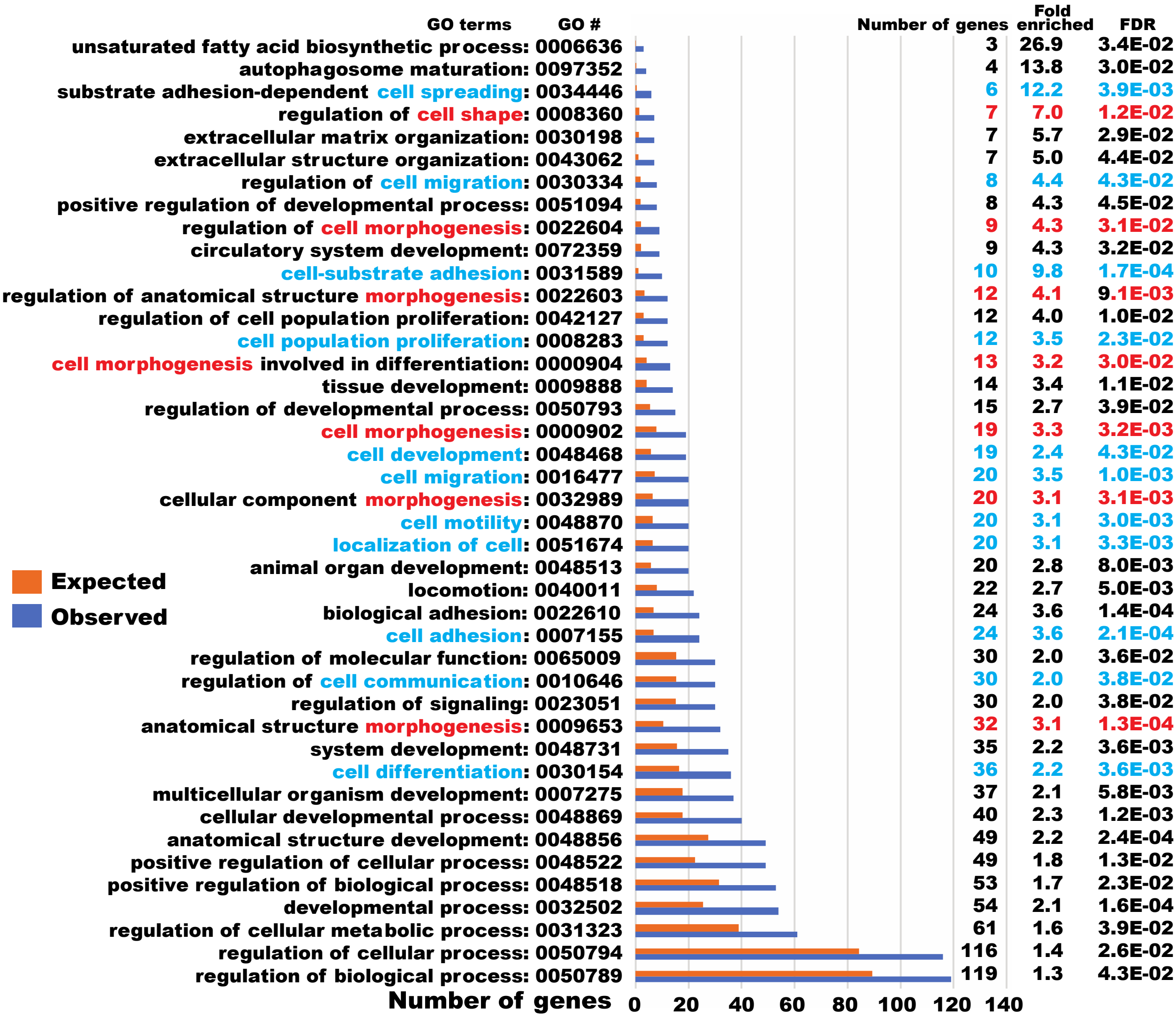

Supplementary Figure S11. Summary for GO analysis of the 449 fibroblast-enriched genes that are resistant to OSKM reprogramming, with the GO annotation data set of “biological process–slim”. GO terms and their associated statistics with the key words of “morphogenesis” and “cell shape” are highlighted in red; those with various types of cell behavior such as “cell spreading”, “cell migration”, “cell communication”, and “cell adhesion” are in cyan.

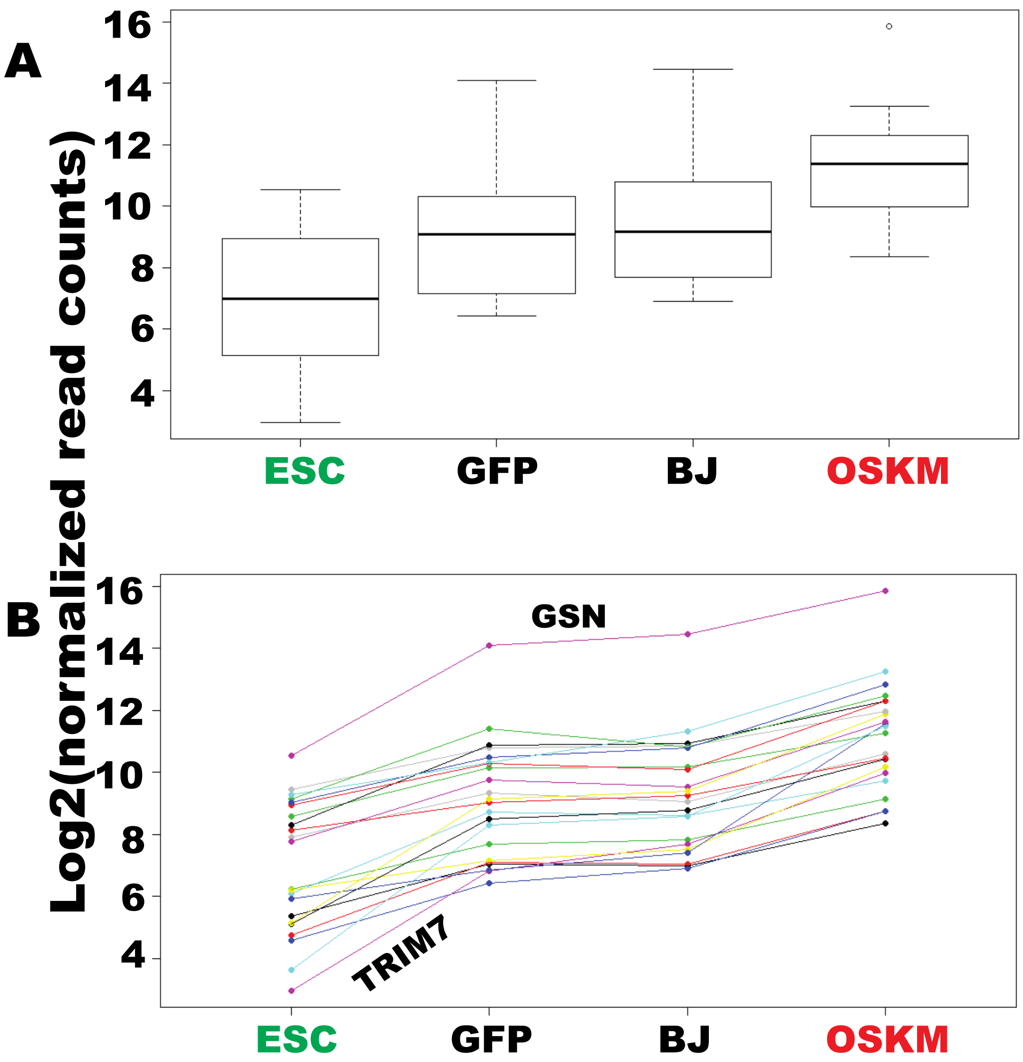

Supplementary Figure S12. 22 genes were wrongly up-reprogrammed. A, A box plot showing that 22 genes were wrongly up-regulated by OSKM when they should be down-reprogrammed. B, A ladder plot for the data set in A to show individually the wrong up-reprogramming of 22 genes. ESC, embryonic stem cells (n = 3); BJ, human fibroblasts (n = 4); GFP, human fibroblasts transduced with GFP lentiviruses (n = 4); OSKM, human fibroblasts transduced with OSKM lentiviruses (n = 2).

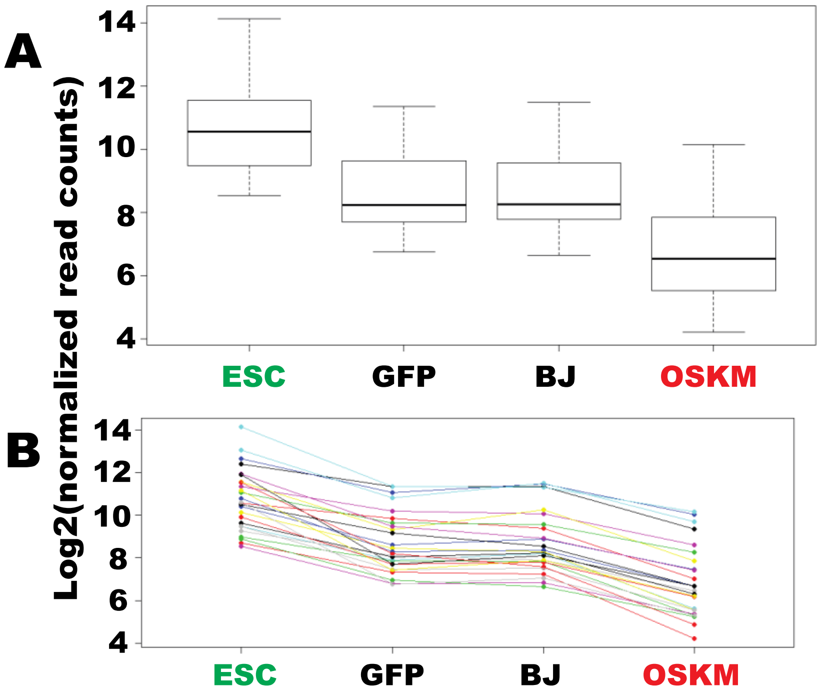

Supplementary Figure S13. 26 genes were wrongly down-reprogrammed. A, A box plot showing that 26 genes were wrongly down-regulated by OSKM when they should be up-reprogrammed. B, A ladder plot for the data set in A to show more individually the wrong down-reprogramming of 26 genes. ESC, embryonic stem cells (n = 3); BJ, human fibroblasts (n = 4); GFP, human fibroblasts transduced with GFP lentiviruses (n = 4); OSKM, human fibroblasts transduced with OSKM lentiviruses (n = 2).

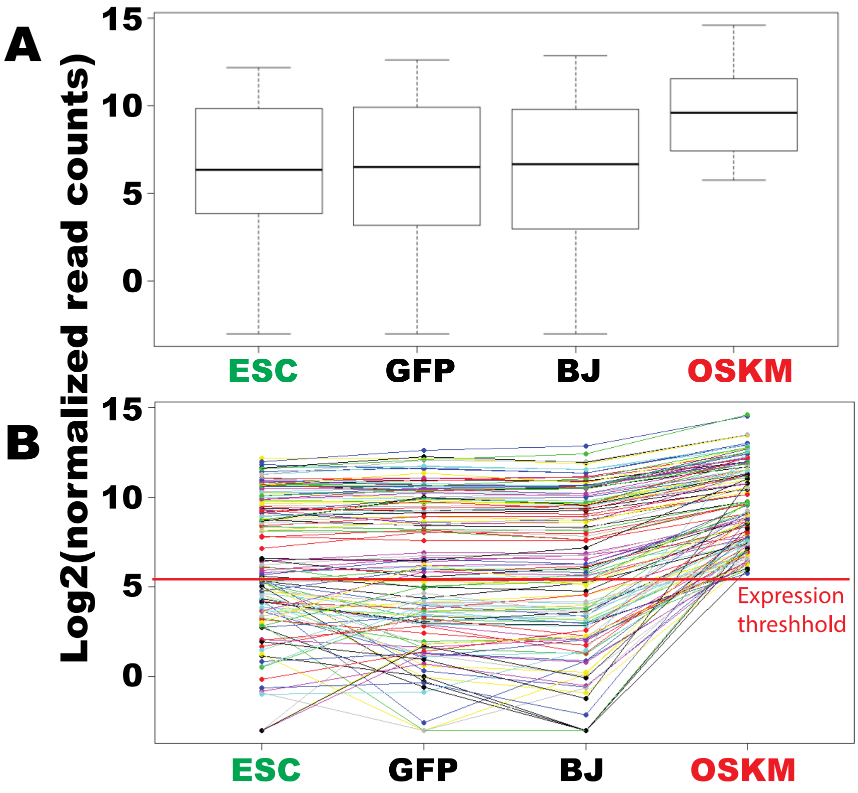

Supplementary Figure S14. 134 genes underwent unwanted up-reprogramming. A, A box plot showing that 134 genes were up-regulated by OSKM when they should not be. B, A ladder plot for the data set in A, but show more individually the unwanted up-reprogramming of 134 genes. ESC, embryonic stem cells (n = 3); BJ, human fibroblasts (n = 4); GFP, human fibroblasts transduced with GFP lentiviruses (n = 4); OSKM, human fibroblasts transduced with OSKM lentiviruses (n = 2).

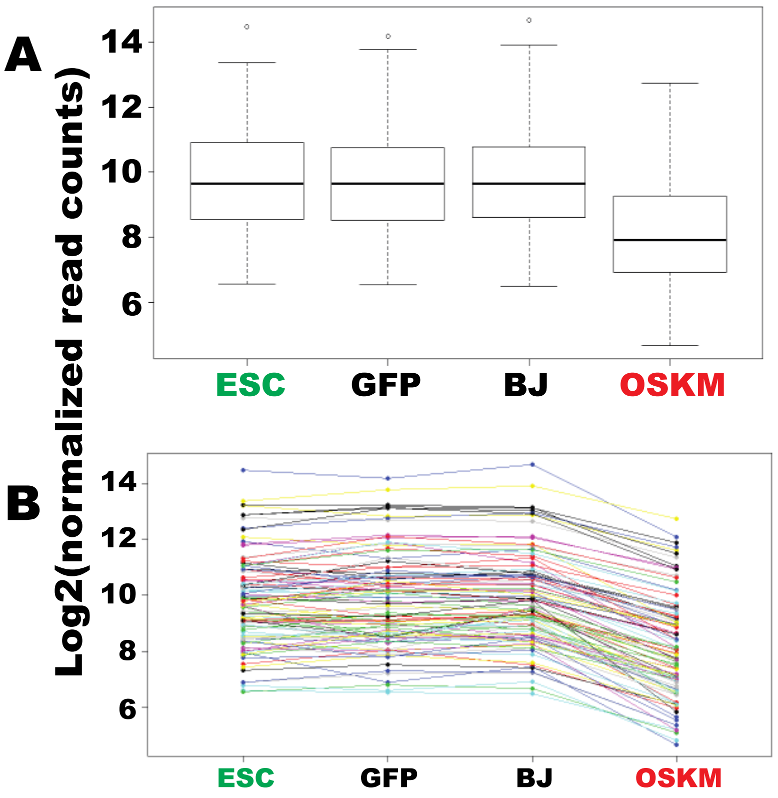

Supplementary Figure S15. 99 genes underwent unwanted down-reprogramming. A, A box plot showing that 99 genes were down-regulated by OSKM when they should not be. B, A ladder plot for the data set in A, but show more individually the unwanted down-reprogramming of 99 genes. ESC, embryonic stem cells (n = 3); BJ, human fibroblasts (n = 4); GFP, human fibroblasts transduced with GFP lentiviruses (n = 4); OSKM, human fibroblasts transduced with OSKM lentiviruses (n = 2).

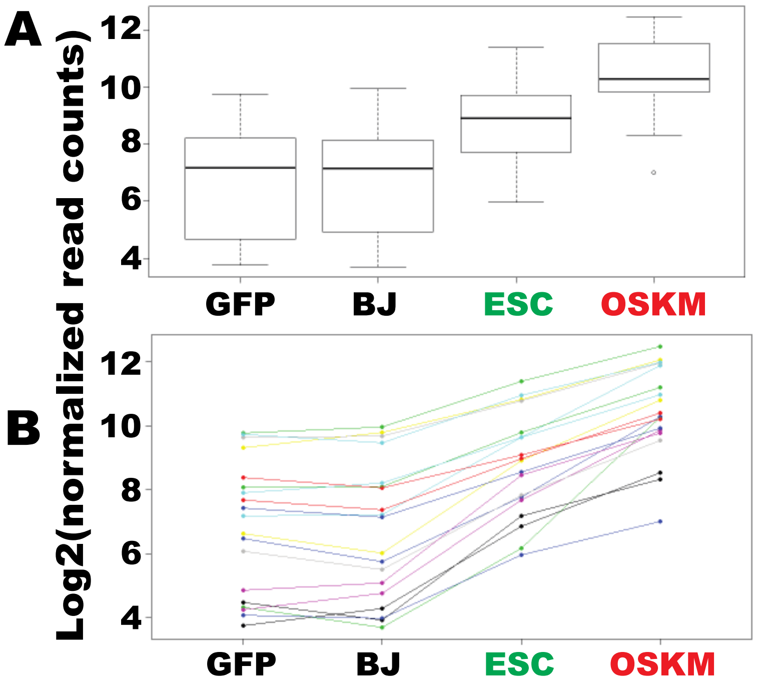

Supplementary Figure S16. 18 genes were over up-reprogrammed. A, A box plot showing that 18 genes as a group were over up-regulated by OSKM. B, A ladder plot showing more individually that 18 genes were over up-regulated by OSKM. ESC, embryonic stem cells (n = 3); BJ, human fibroblasts (n = 4); GFP, human fibroblasts transduced with GFP lentiviruses (n = 4); OSKM, human fibroblasts transduced with OSKM lentiviruses (n = 2).

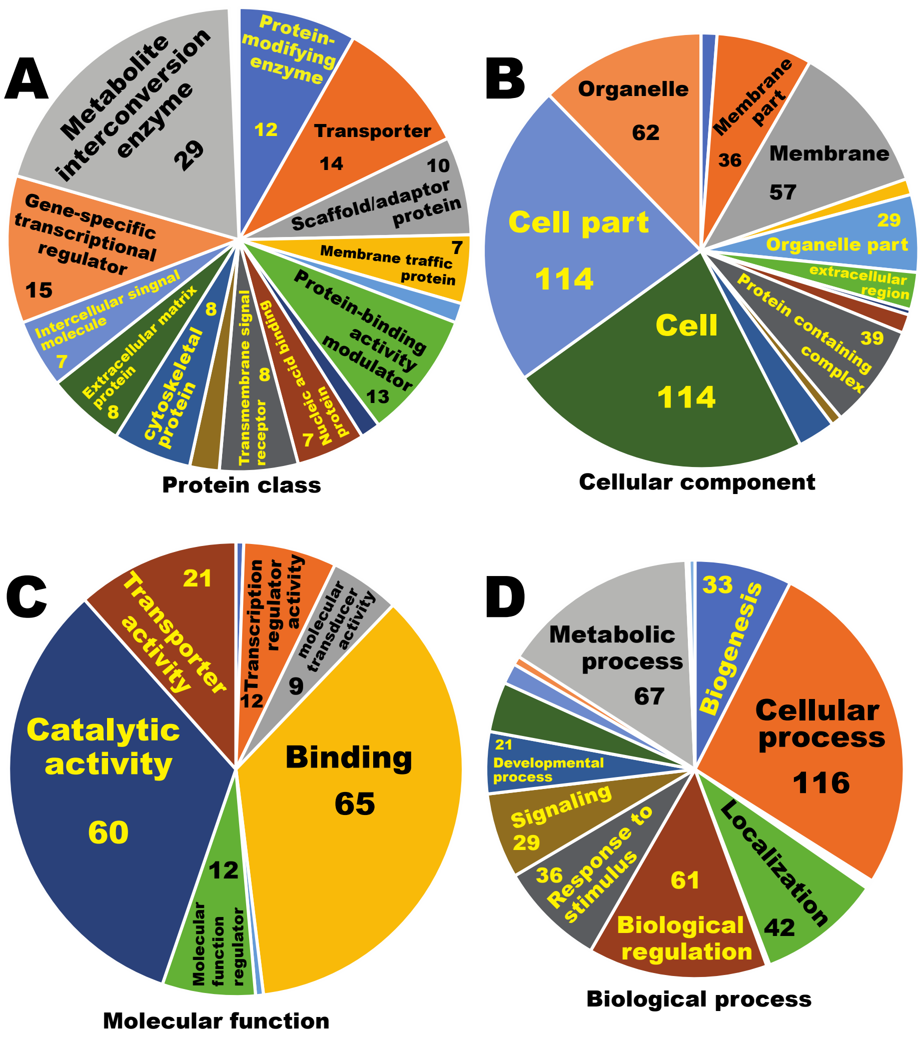

Supplementary Figure S17. Pie-chart summary of GO classification analyses with the 305 aberrantly reprogrammed genes. A, protein class classification. B, Cellular component classification. C, Classification based on molecular function. D, Classification based on biological process. Numbers in each pie section is the amount of genes that is placed in the corresponding category by GO classification.
